# Supplementary figures and images for: Optimal performance of stand-alone hybrid microgrid systems based on integrated techno-economic-environmental energy management strategy using the grey wolf optimizer
Source: PLoS One. 2024 Feb 8;19(2):e0298094. doi: 10.1371/journal.pone.0298094 (PMC10852242; doi:10.1371/journal.pone.0298094)

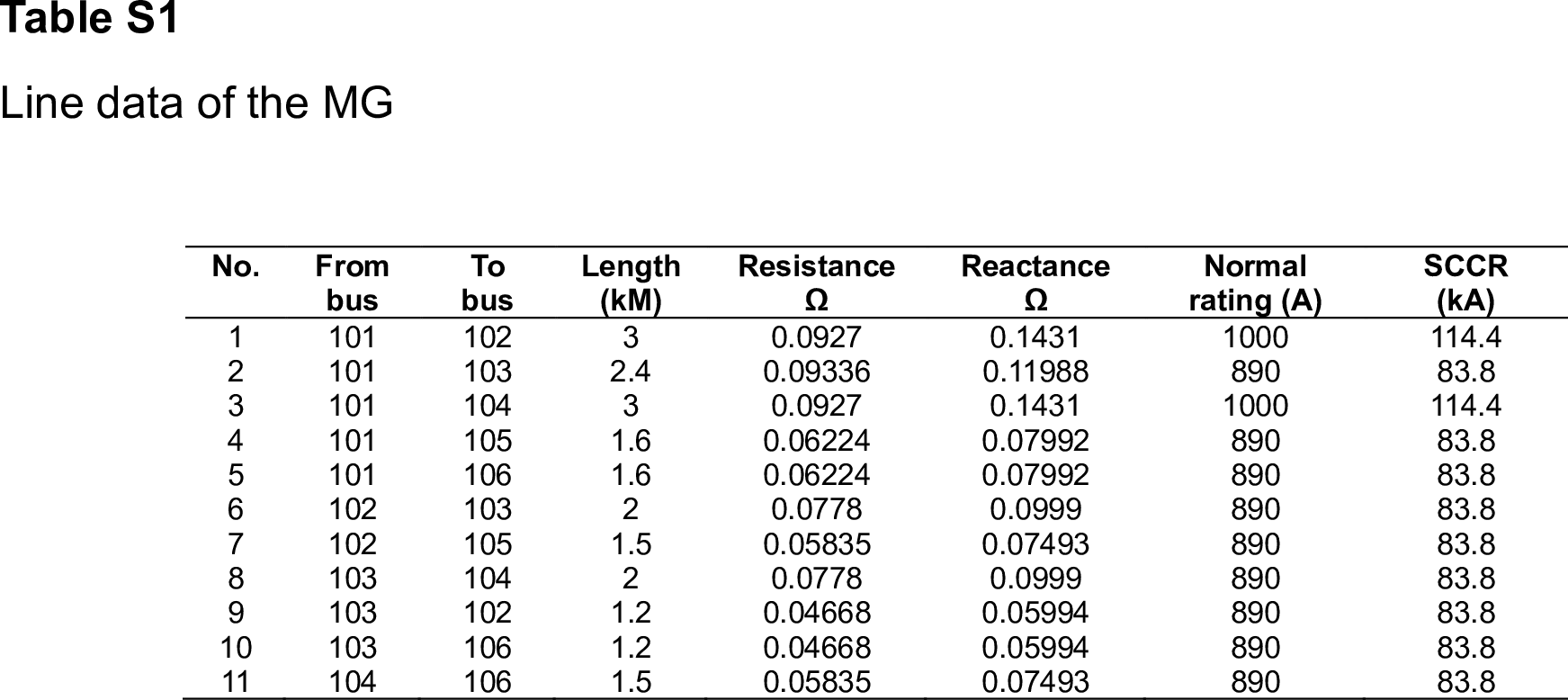

Supplement: S1 Table — (TIF) [file pone.0298094.s001.tif]

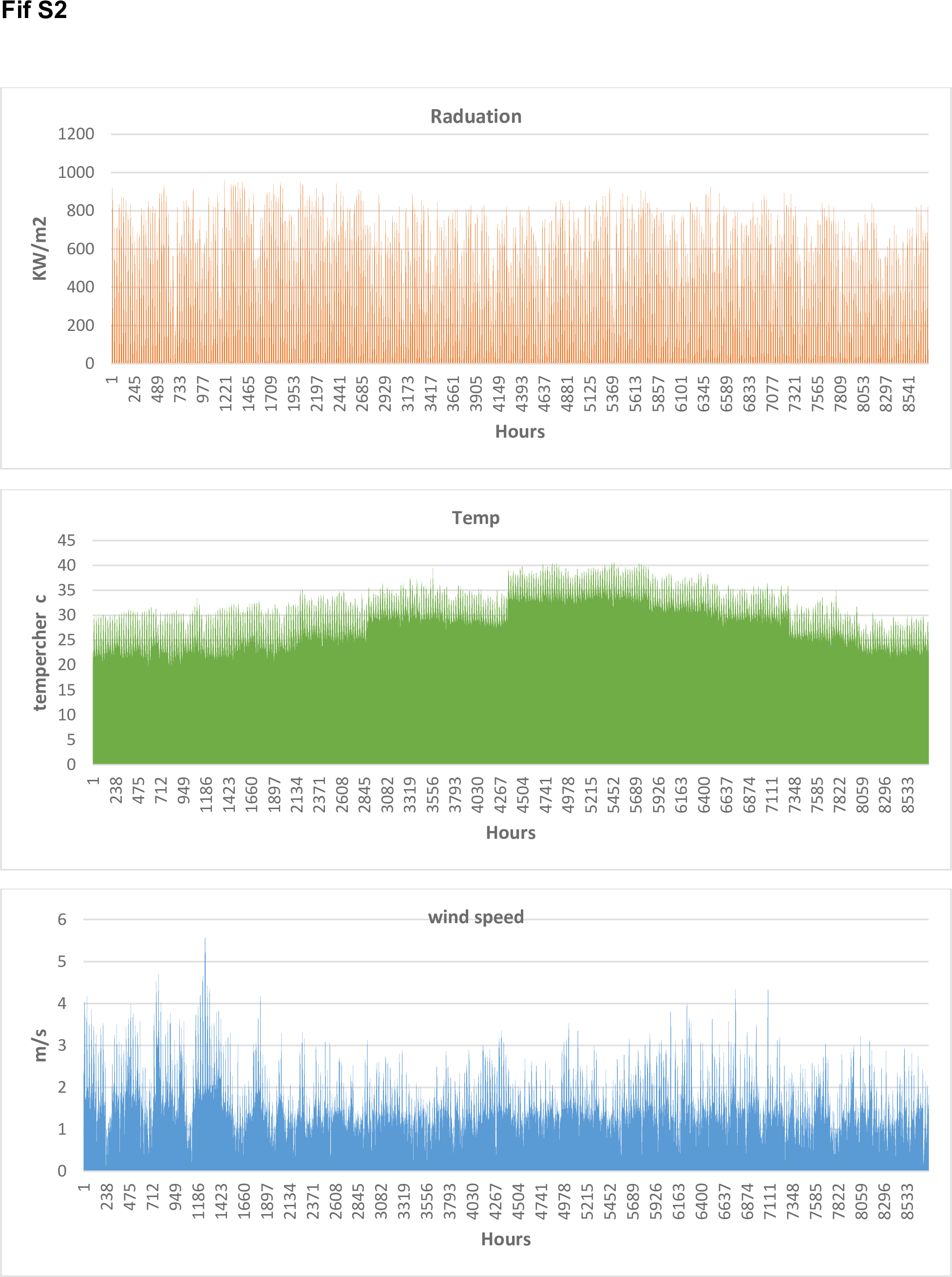

Supplement: S1 Fig — (TIF) [file pone.0298094.s002.tif]
