# Supplementary material for: Optimal performance of stand-alone hybrid microgrid systems based on integrated techno-economic-environmental energy management strategy using the grey wolf optimizer
Source: PLoS One. 2024 Feb 8;19(2):e0298094. doi: 10.1371/journal.pone.0298094 (PMC10852242; doi:10.1371/journal.pone.0298094)
Supplement: Attachment — Submitted filename: Response to reviewers.pdf [file pone.0298094.s003.pdf]

PLOS ONE Decision: Revision required [PONE-D-23-29832] - [EMID:c79f2e72a4d8c137]

Dear Sir

I hope this message finds you well. I would like to express my gratitude to you and the reviewers for taking the time to evaluate my manuscript titled " **Optimal performance of stand-alone hybrid microgrid systems based on integrated techno-economic-environmental energy management strategy using the grey wolf optimizer**" I appreciate the constructive feedback and insightful comments provided during the review process.

I have carefully considered each of the reviewers' comments and suggestions, and I am pleased to inform you that I have made the necessary revisions to address their concerns. I believe these changes have significantly strengthened the quality and clarity of the manuscript.

In response to Reviewer 1's comments regarding [**Figure 1 in our submission contain copyrighted images**], I have removed the copyrighted images from Figure 1 and replaced them with illustrations prepared by the authors. Additionally, I have implemented all the magazine's requirements mentioned in the revisions report. I believe these revisions have enhanced the overall coherence and impact of the paper.

I am confident that the revised manuscript now meets the high standards of PLOS ONE, and I hope it will be suitable for publication. I have attached the revised version for your consideration.

Once again, thank you for the valuable feedback and guidance provided by the reviewers. I appreciate the time and effort invested in the review process.

I look forward to hearing from you soon.

Best regards,

Tukkee Ahmed Sahib
